# Toujie Quwen granule used with conventional western therapy for coronavirus disease 2019: A protocol for systematic review and meta-analysis

**DOI:** 10.1097/MD.0000000000026370

**Published:** 2021-06-18

**Authors:** Peng Sun, Dongmei Yan, Bin Li, Liping Tang, Lingxia Xu, Fei Wang

**Affiliations:** a The Affiliated Hospital of Jiangxi University of Chinese Medicine; b Academician Workstation, Jiangxi University of Chinese Medicine, Nanchang, Jiangxi, China.

**Keywords:** Coronavirus disease 2019, protocol, systematic review, Toujie Quwen granule

## Abstract

**Background:**

Coronavirus disease 2019 (COVID-19) is an epidemic infectious disease resulted from 2019 novel coronavirus (2019-nCoV). Up till now, COVID-19 has swept globally. Currently, due to many high-profiled benefits, clinical studies on Toujie Quwen granule (TJQW) have been increasing. The aim of the study is to assess the efficacy and safety of TJQW used with conventional western therapy for COVID-19.

**Methods:**

Relevant randomized controlled trials (RCTs) were searched in Chinese and English databases, and the search time is January 2020 to May 2021. English databases include PubMed, Embase, Web of Science, and the Cochrane Library. Chinese databases include CNKI, WF, VIP, and CBM. The international clinical trial registration platform and the Chinese clinical trial registration platform of controlled trials will be searched by us from January 2020 to May 2021. According to the inclusion and exclusion criteria, screening literature, extraction data will be conducted by 2 researchers independently. Statistical analysis will be conducted using the RevMan 5.3.5 software. After screening the literature based on the inclusion and exclusion criteria, The Recommendation, Assessment, Development, and Evaluation (GRADE) system will be used to evaluate the quality of each result.

**Results:**

This study will provide the evidence for TJQW to be used with conventional western therapy for COVID-19.

**Conclusion:**

The efficacy and safety of TJQW used with conventional western therapy for COVID-19 will be assessed.

**INPLASY registration number:**

INPLASY202150038

## 1 Introduction

Coronavirus disease 2019 (COVID-19) is an epidemic infectious disease resulted from 2019 novel coronavirus (2019-nCoV).^[1,2]^ COVID-19 has unique characteristics, such as strong infectivity, long incubation period, various clinical manifestations, and wide susceptible range.^[3]^ Up till now, COVID-19 has swept globally.^[4]^ Based on the symptom, COVID-19 include mild, ordinary, severe, and critical types.^[5]^ The clinical manifestations of patient include fever, cough, nasal congestion, runny nose, diarrhea, dyspnea, muscle or joint pain, pneumonia, and even death.^[5]^ Currently, the therapies recommended by western clinical practice guidelines for COVID-19 include antiviral, oxygen therapy, and nutritional support, however, no specific and effective drug is available for COVID-19.^[6]^

In China, Chinese traditional medicine is recommended by the Chinese Clinical Guidance of COVID-19 Pneumonia Diagnosis and Treatment (7th edition) published by China National Health Commission on March 4, 2020, which includes Xuebijing injection, lianhua qingwen granules, and jinhua qinggan granules.^[7]^ Moreover, since the spread of COVID-19, Chinese medicine hospital of every province recommended unique TCM formulations for COVID-19 treatment, for example, Toujie Quwen granules (TJQW).^[8–12]^ TJQW includes 16 TCM components, including Forsythiae Fructus (lian-qiao), edible tulip (shan-ci-gu), *Lonicera japonica* (Japanese honeysuckle flower, jin-yin-hua), Radix Scutellariae baicalensis (huang-qin), Folium Isatidis (da-qing-ye), Bupleurum root (chai-hu), Artemisia apiacea (qing-hao), Periostracum Cicadae (chan-tui), Radix Peucedani (qian-hu), *Fritillaria cirrhosa* (chuan-bei-mu), *Fritillaria thunbergii* (zhe-bei-mu), Poria cocos (fu-ling), Fructus Mume (wu-mei), radix Scrophulariae (xuan-shen), *Astragalus propinquus* (huang-qi), and radix Pseudostellariae (tai-zi-shen).^[8–12]^ In addition, network pharmacology studies have demonstrated that therapeutic mechanism of TJQW in COVID-19, and TJQW treatment for COVID-19 was associated with the improvement of immunity and suppression of inflammatory stress, including regulation of inflammatory response, viral process, neutrophil mediated immunity, PI3K-Akt signaling pathway, MAPK signaling pathway, Jak-STAT signaling pathway, complement and coagulation cascades, and HIF-1 signaling pathway.^[12]^

However, the systematic review examining the efficacy and safety of TJQW in the treatment of COVID-19 is absent. Therefore, the purpose of this study is to explore the efficacy and safety of TJQW in the treatment for COVID-19 by pooling the current randomized controlled trials, in order to provide the high-quality clinical evidence.

## 2 Methods

This is a systematic review and ethical approval was not necessary.

### 2.1 Study registration

This systematic review protocol has been registered on INPLASY website and registration number were INPLASY202150038 (https://www.doi.org/10.37766/inplasy2021.5.0038).

### 2.2 Database search

Relevant randomized controlled trials (RCTs) will be systematically searched from 4 English medical databases (PubMed, Embase, Web of Science, the Cochrane Library) and 4 Chinese medical databases (CNKI, VIP, CBM, WF database), and the search time will be January 2020 to May 2021. The search strategy will be based on the guidance of the Cochrane handbook. The search strategy for PubMed is shown in Table 1.

**Table 1:** Search strategy used in PubMed database.

Number	Search terms
1	New coronavirus[mh]
2	Novel coronavirus [mh]
3	COVID-19 [mh]
4	2019-nCoV [mh]
5	SARS-CoV-2 [mh]
6	or 1–5
7	randomized controlled trial [pt]
8	controlled clinical trial [pt]
9	randomized [tiab]
10	randomly [tiab]
11	or 7–10
12	animal[mh]
13	humans[mh]
14	12 not 13
15	Toujie Quwen[mh]
16	6 and 11 and 14 and 15

### 2.3 Eligibility criteria

#### 2.3.1 Type of study

In this study only RCTs will be eligible for inclusion.

#### 2.3.2 Types of participants

The eligible patients with confirmed COVID-19 according to the “New Coronavirus Pneumonia Diagnosis and Treatment Program” (trial seventh edition).

#### 2.3.3 Types of interventions

##### 2.3.3.1 Experimental interventions

The experimental interventions were treated with TJQW combined basic treatment.

##### 2.3.3.2 Control interventions

The control group include antiviral, oxygen therapy, and nutritional support.

#### 2.3.4 Outcomes

##### 2.3.4.1 Primary outcomes

The primary outcome measurements include the cure, aggravation, and mortality rate.

##### 2.3.4.2 Secondary outcomes

Secondary outcomes are as follows: the recovery rate of fever; the recovery rate of cough; the recovery rate of fatigue; the duration of fever; the duration of cough; the duration of fatigue; negative conversion rate of nucleic acid test; improvement or recovery of chest CT manifestations; length of hospitalization; adverse events.

### 2.4 Exclusion criteria

1. The unrelated documents and studies repeated publication will be deleted.
2. Semi-randomized controlled trials, retrospective studies experience summaries, case series, case reports, animal experiments, and reviews.
3. Review articles without incomplete data and have obvious errors.

### 2.5 Data screening and extraction

#### 2.5.1 Data screening

**Figure 1:**
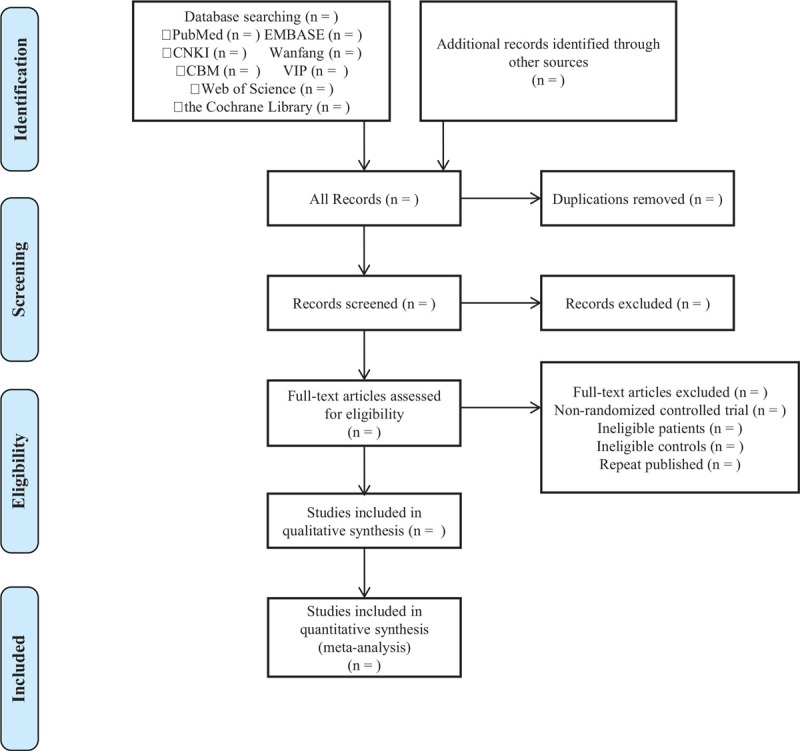
PRISMA-P flow chart.

Uploading the eligible studies searched into the document management software of Endnote X9. Two independent researchers will screen the literature according to the eligibility criteria and exclusion criteria. When there is inconsistent opinion, 3 researchers will determine whether the articles can be ultimately included. Details of the selection procedure for studies are shown in a PRISMA-P flow chart (Fig. 1).

#### 2.5.2 Data extraction and management

The following information from selected studies will be summarized in a unified table: basic information of the ultimate eligible studies: first author's last name, time of publication, first author's country, diagnostic criteria, inclusion criteria, exclusion criteria, sample size; baseline characteristics of the patients: mean baseline age, the level of severity of COVID-19, basic therapies; treatment and control measurements: dose of TJQW, course of treatment; outcomes; adverse events.

### 2.6 Quality assessment

Risk of bias for the included RCTs will be assessed by Review Manager 5.3. Evaluation items are as follows: whether random sequences are generated; whether distribution is hidden; whether participator and researchers are blinded; whether study outcomes are blinded; whether outcome data exist missing; whether reporting is selective; other sources of bias. Summary of each item results with a high, low, or unclear risk of bias will be displayed as a table. Two investigators will independently assess the quality of the included studies, and disagreement in risk of bias will be resolved by 3 investigators through discussions.

### 2.7 Statistical analysis

#### 2.7.1 Data analysis and processing

RevMan 5.3 will be used for data analysis. Odds ratio (OR) or relative risk (RR), 95% confidence interval (CI), and *P* values will be used to estimate dichotomous variables include. The continuous data will be analyzed by mean difference (MD) or standard mean difference (SMD), 95% confidence interval (CI), and *P* values. Concurrently, the *Q* value test and *I*^2^ index will be used to measure the statistical heterogeneity. The meta-analyses will be based on the random effects model, otherwise, a fixed effect model will be adopted.

#### 2.7.2 Subgroup analysis

If we find substantial heterogeneity, the grouping factor for subgroup analysis will be executed according to the level of severity of COVID-19 and age of patient.

#### 2.7.3 Sensitivity analysis

Sensitivity analysis will be performed by excluding studies with high risk of bias and change the statistical model.

#### 2.7.4 Publication bias

When ≥10 studies are included, we will use a funnel plot to assess publication bias. However, due to the limitations of a funnel plot, Egger test will be used to help assess publication bias. The analysis software is STATA 3.5.1 for Windows.

#### 2.7.5 Quality of evidence

The included literature evaluates the evidence in the light of GRADE method, the levels of quality of evidence will be ranked as 4 levels: high, moderate, low, and very low. All processes are on this page: https://gradepro.org/

### 2.8 Ethics and dissemination

This systematic review protocol does not involve any intervention or breach of personal privacy; thus, ethical review is not needed. We aim to publish the results in a peer-reviewed journal.

## 3 Discussion

At present, COVID-19 has swept the world, and has become a global problem that all health organizations in the world continue to pay attention to.^[13]^ In China, early intervention and prevention of traditional Chinese medicine and integrated treatment of traditional Chinese and Western medicine have become one of the important approaches to cure patients with COVID-19.^[14]^ Therefore, it is necessary to evaluate the efficacy and safety of TJQW in the treatment of COVID-19 to better guide the clinical application of TJQW in the treatment of COVID-19, which will provide a basis for better treatment options.

### 3.1 Limitations

There are language restrictions in this study, inclusion of Chinese and English papers, which means there is a risk of bias. Because there are many kinds of TCM preparations, heterogeneity risk may increase. In addition, the standard of judging the level of evidence has not been standardized.

## Author contributions

**Conceptualization:** Peng Sun, Lingxia Xu.

**Funding acquisition:** Peng Sun, Dongmei Yan, Fei Wang.

**Investigation:** Peng Sun, Dongmei Yan, Liping Tang.

**Methodology:** Lingxia Xu.

**Supervision:** Bin Li.

**Writing – original draft:** Peng Sun, Dongmei Yan, Liping Tang, Lingxia Xu.

**Writing – review & editing:** Peng Sun, Lingxia Xu, Fei Wang.
